# Supplementary material for: Neural network-based Bluetooth synchronization of multiple wearable devices
Source: Nat Commun. 2023 Jul 25;14:4472. doi: 10.1038/s41467-023-40114-2 (PMC10368670; doi:10.1038/s41467-023-40114-2)
Supplement: Supplementary file 1 — Supplementary Information [file 41467_2023_40114_MOESM1_ESM.pdf]

Supplementary Information

Neural network–based Bluetooth synchronization of multiple wearable devices

Karthikeyan Kalyanasundaram Balasubramanian[1], Andrea Merello[1], Giorgio Zini[1], Nathan Charles Foster[2], Andrea Cavallo[2,3], Cristina Becchio[4, 2], and Marco Crepaldi[1]

[1] Electronic Design Laboratory (EDL), Istituto Italiano di Tecnologia, Genova, Italy
[2] Cognition, Motion and Neuroscience (C'MON), Istituto Italiano di Tecnologia, Genova, Italy
[3] Department of Psychology, University of Turin, Torino, Italy
[4] Department of Neurology, University Medical Centre Hamburg-Eppendorf, Hamburg, Germany

Supplementary Note 1: Wearables design methodology

Acquiring health data has been a demanding criterion for human welfare in the last few decades. Its principal driver is wearable devices today, which have advanced systematically in productivity and performance in multiple manifolds while being versatile and portable. Technological factors that must be contemplated while designing such wearable devices typically depend on (i) Wearability: comfort, color, portability, and compactness can impact participants' physical attention and remain critical physical constraints among wearables. (ii) Battery life: power management is becoming increasingly relevant for carrying out fundamental routines and specific tasks without interruption. Furthermore, various aspects, such as efficient coding, data packing, encryption, and compression, can boost computational power. (iii) Connectivity and data flow management: the host (e.g., laptop or tablet) can instantly connect to the wearables for high-level data management, i.e., managing the three levels of data flow: data acquisition, transmission to the host, and storage and retrieval, while the device connection is secure and the data is encrypted. (iv) Data security: often, sensitive data is collected, stored, and processed. Thereby ensuring security measures and end-to-end encryption remains obligatory. (v) Interoperability: the design meets industry standards and protocols so that the device can communicate seamlessly with other devices and systems. (vi) Sustainability: minimizing waste and maximizing efficiency while considering the environmental impact during device conception. Reusing wearables with new programming techniques could help improve or facilitate additional features without the need for new devices, supporting sustainability.

Cloud or edge devices for health care

Consumer devices from the wearable devices category, such as a smartwatch, provide notable features (for instance, the possibility of combining multiple sensor inputs, cloud, and internet connectivity, significant computing power, and advanced graphics) and can perform admirably in the field of sensing computing and communication convergence. Notwithstanding, they practically fail in specialized stand-alone applications where real-time data acquisition is paramount due to their by-design general-purpose features and form factor, involving *inter-alia*, data security, internet connectivity, and aesthetics (that can predominantly distract human attention[1]). On the other hand, edge devices with essential functions (i.e., a computing device with a battery, sensor, storage device, and connectivity) are the perfect solutions for wearables in such specialized assessments[2–6] in health care.

Supplementary Note 2: KiD Hardware layout

KiD comprises a commercial `Microcontroller` unit (STM32L476[7], ST Microelectronics), a `Bluetooth` Low Energy module (BLE, BGM123A[8], Blue Giga that supports BLE 4.2 specification[9], and works as a network co-processor[10]), a Lithium Polymer (LiPo) `Battery Controller` (BQ24230[11], Texas Instruments), an `Inertial Measurement Unit` (IMU, MPU-9250[12], Invensense) that consists of a nine-axis (gyroscope, accelerometer compass), and an onboard Quad Serial Peripheral Interface (Quad-SPI) `Flash memory` (S25FL256SAGNFI001[13], Spansion/Cypress) as shown in Supplementary Fig. 1a. In particular, a Real-Time Operating System (RTOS, here we use FreeRTOS: `https://www.freertos.com`) that runs in the `Micro controller` of the wearable device to actively manage and operate with the connected hardware components to implement routine tasks to function as a standalone system. A `Battery controller` regulates the battery charging and maintains a constant 3.3 V voltage supply for all the individual hardware components in the device. KiD can exchange data in two different modes: i) using the `Bluetooth` (BLE using Generic Attribute Profile, GATT) that receives the wireless commands (see Supplementary Table 1 for the KiD user-level commands) from the experimenter and control the data path of the `Microcontroller` accordingly, and ii) using the `Micro USB` port, to perform large data transfer (transmission of captured motion profiles to PC). Moreover, the KiD can use the `Micro USB port` for charging the battery. The IMU inside this device has a significant measurement range, that is $\pm$ 4G ($\pm$ 39.23 ms^2) for the accelerometer, $\pm$2000 $^\circ s^{-1}$ for the gyroscope and $\pm$ 4912 μT for the compass. The internal `Flash memory` (256 Mb) stores the inertial data from the IMU in a pre-defined format (see Supplementary Table 2). All these components are operated by a LiPo battery of 100 mAh capacity packed inside the `Plastic inner case`, resulting in a continuous operation time of approximately 2.5 hours, available to perform motion capture experiments (see Supplementary Fig. 1b).

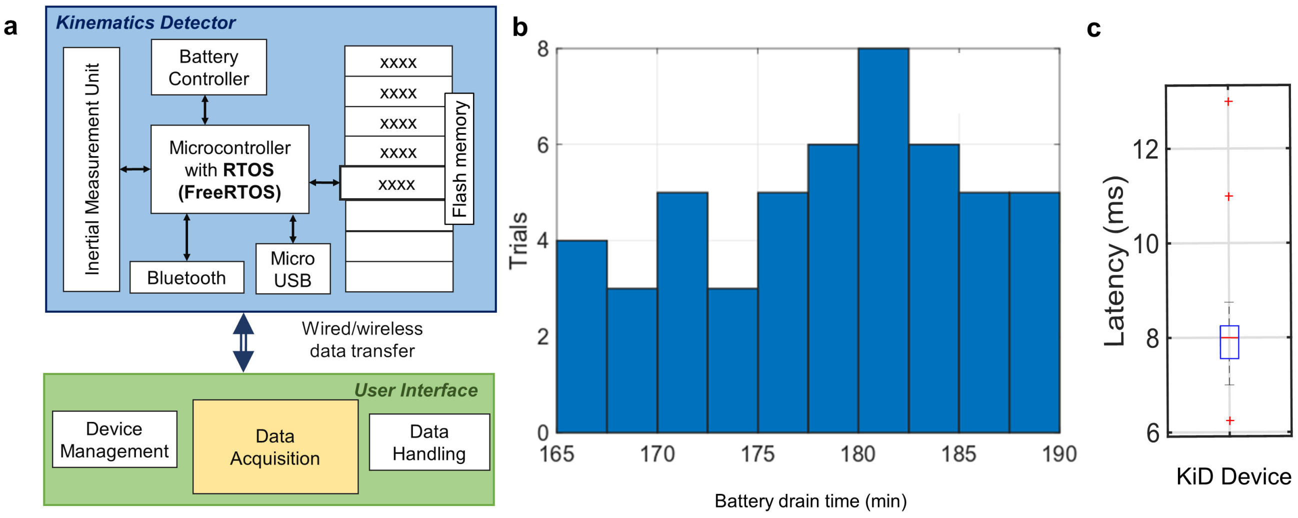

Supplementary Figure 1. a, KiD with the hardware components that has an inbuilt `Flash memory`. **b**, Latency of the KiD when operated as a single device (the mean value found to be KiD 8 ms). **c**, The battery drain time is the maximum battery retention in KiD with a 100 mAh battery (the mean value found to be 179$\pm$7 minutes).

Structure of RTOS, the firmware

RTOS does active multitasking and pre-emptive task scheduling, essentially permitting low-power management. The RTOS task scheduler is tightly coupled with hardware components to execute tasks synchronously or asynchronously, and its operation strongly depends on the device's Real-Time Clock (RTC). Each component in the hardware is conceptually classified (systematically probed by specific functions) and methodically prioritized to implement KiD functionalities. For instance, an IMU is partitioned into several runtime tasks, such as initialization, mode selection, filtering characteristics, and data transfer, to achieve motion capture at a high level. KiD comprises a few device commands (i.e., programmed using C++™) representing some primitive operations to define the `Microcontroller` internal data path. `START` command will initiate motion profile acquisition, `MARK` adds a unique label directly into the device's internal memory, and `STOP` stops motion capturing (see Supplementary Table 1). Every received command is processed and acknowledged instantaneously to update as soon as possible the device status. Moreover, KiD has a system minimal latency of 8 ms (see Supplementary Fig. 1c), making it highly responsive.

Supplementary Table 1. KiD user-level commands.

Command	Details	Synchronous or Asynchronous message	Response from KiD
START	It starts the motion acquisition	Synchronous	Ok or Error
STOP	It stops the motion acquisition		
MARK	It sends an online label		
TIME	It returns the device time	Asynchronous	Time or Error
VOLT	It returns the device battery level		Battery Level or Error
ERASE	It erases the device memory		End or Error
DWLD*	It downloads the device content to PC		Ok or Error
* command to be used only when the device is connected with USB – Universal Serial Bus			

Supplementary Table 2. A typical motion profile with an identifier and motion data.

Accelerometer			Gyroscope			Compass			Quaternions				Mark
**AX**	**AY**	**AZ**	**GX**	**GY**	**GZ**	**CX**	**CY**	**CZ**	**QW**	**QX**	**QY**	**QZ**	**mark**
**Motion data**													
515	-3998	6860	83	11	11	228	275	-98	10283482	-30302270	-35967282	37157891	0
276	-4109	7504	73	15	55	229	258	-98	10283482	-30412013	-35942343	382829242	0
**Identifier**													
0	0	0	0	0	0	0	0	0	0	0	0	0	134

KiD's motion profile

`Motion data` is the kinematic information of a limb activity at a certain instant (see Supplementary Table 2). The `Motion data` is a direct component from the IMU, sampled at a given rate (200 Hz), and it contains a tri-axis accelerometer, gyroscope, compass, and Quaternions data (a scalar and vector component of orientation in a complex space). A motion profile is the accumulation of these `Motion data`. The `Identifier` in a motion profile does not provide `Motion data`, but rather it provides a unique label (marker to identify a task).

Supplementary Methods: KiD Software flow chart

The User Interface (UI) is designed with Python™ 3.8 programming language, and Qt™ 5.2 (a cross-platform software development Framework). Python provides exceptional power in managing, interfacing, and driving the hardware and software[14].

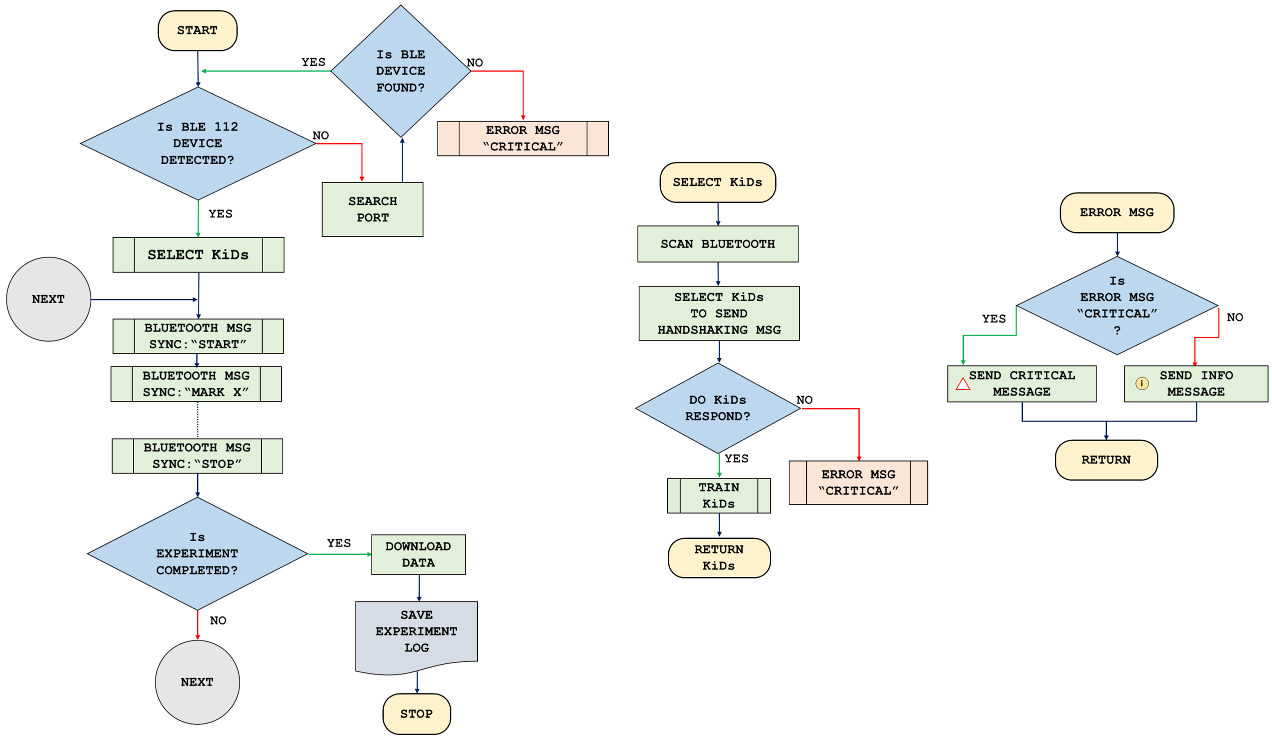

Supplementary Figure 2. Flow chart that shows the functionality of the user interface, with error messages and status messages.

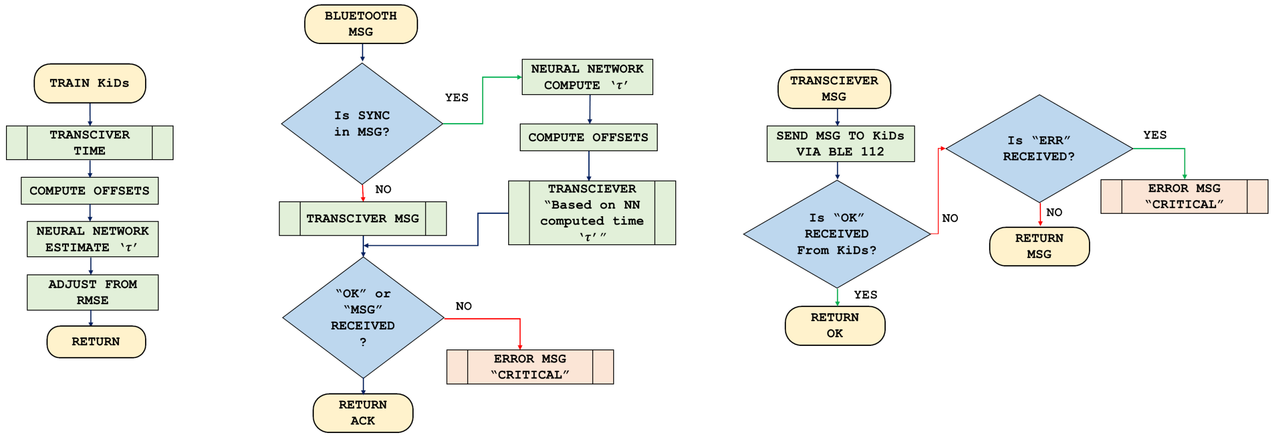

Supplementary Figure 3. Flow chart that shows how the information or data are sent to the KiD from the Remote system.

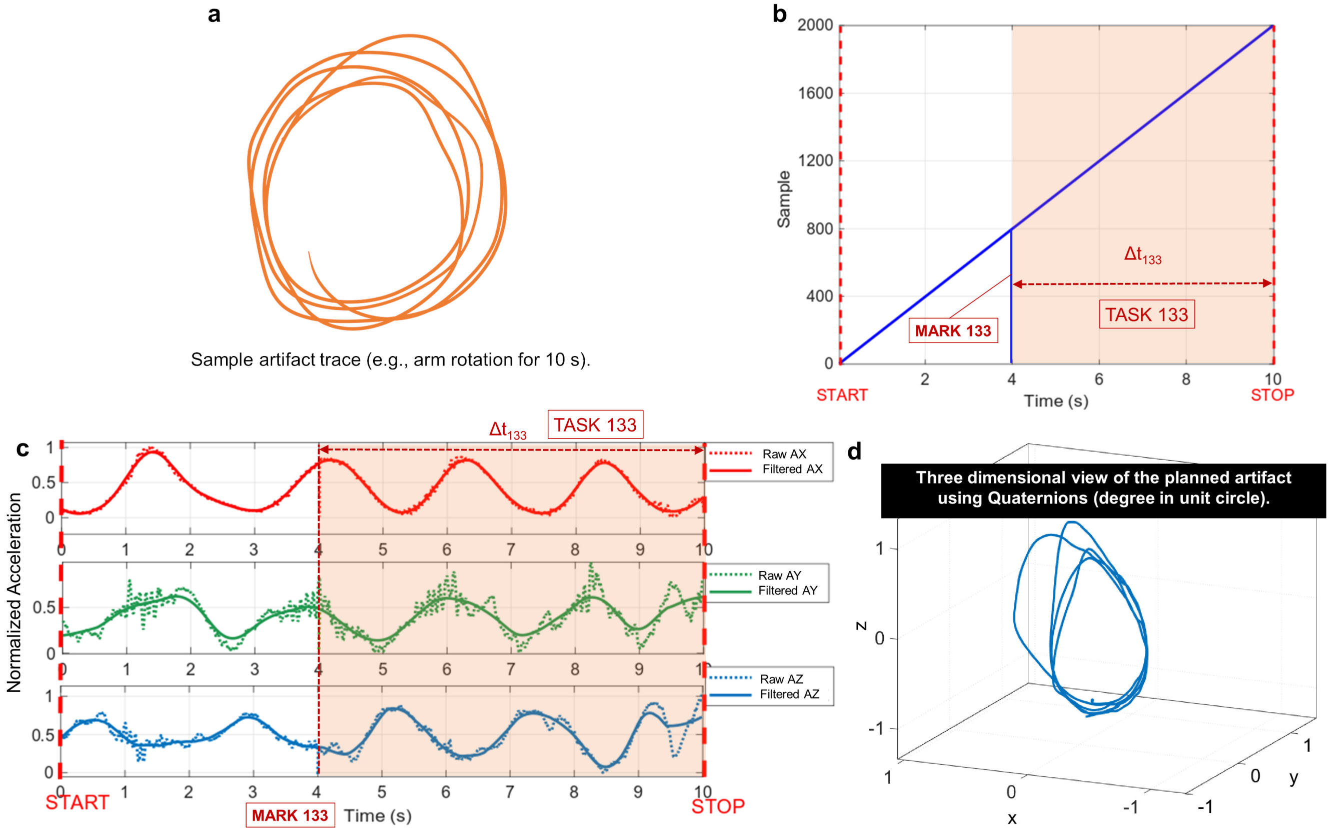

Supplementary Figure 4. **a** Sample artifact trace of a hand rotation. **b** Timeline in the motion profile show how `MARK 133` inserts the `Identifier` to the KiD's onboard memory. The colored window shows the `TASK 133` as an identified task in the motion profile. **c** Accelerometer readings (`AX`, `AY`, and `AZ`) from the motion profile. **d** Three-dimensional view of the motion profile using Quaternions.

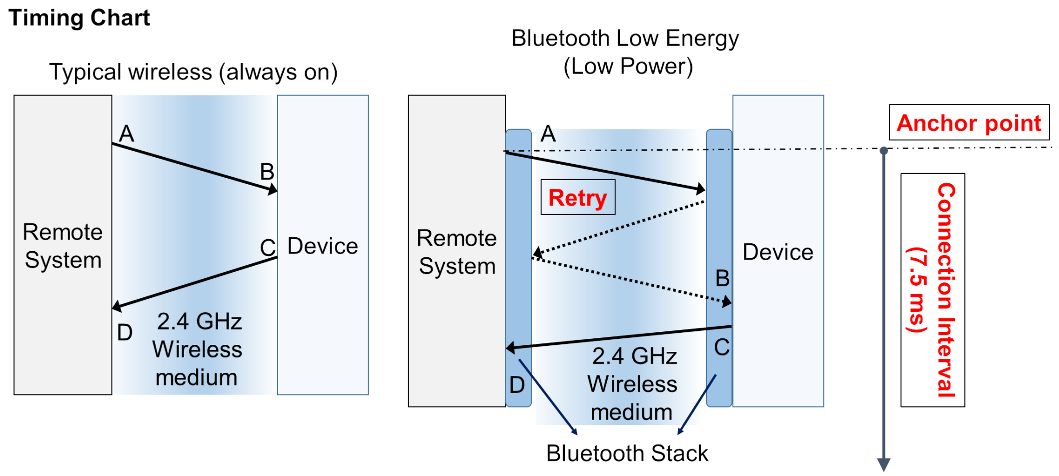

Supplementary Figure 5. Working of the Bluetooth Low Energy compared to the other wireless technology. The Bluetooth stack in the remote system implements a `Retry` of packet transmission until it receives a suitable acknowledgment.

Supplementary Methods: Neural network concepts

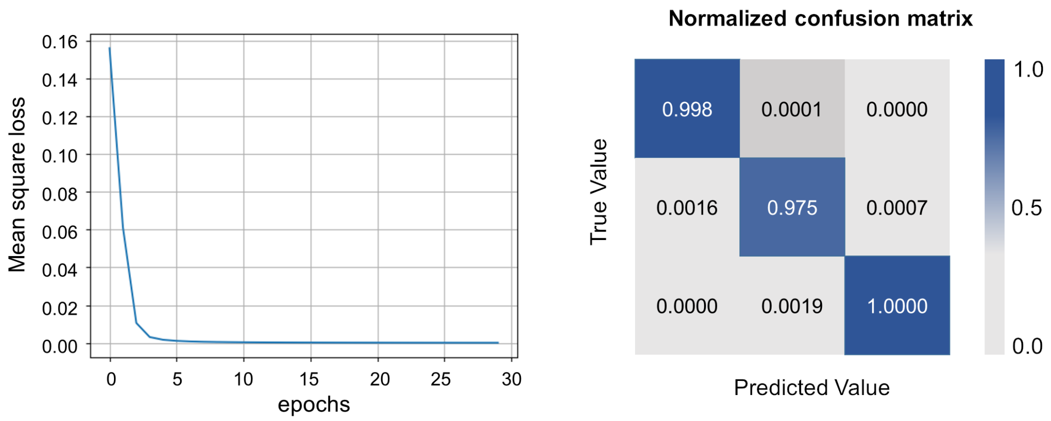

Supplementary Figure 6. Neural network training parameters: mean square error with epochs, and confusion matrix to classify the predicted value with actual value.

Here the device works on a Neural network. We demonstrate the network using TensorFlow. Fig. 6 shows the loss while training the network and the confusion matrix between the actual and predicted values for the simulated neural network. The designed model/network is further integrated into the UI to act as a virtual clock, thus predicting the wearable devices' time value to perform mutual synchronization.

Supplementary Discussion: Motor behavior or sensorimotor analysis –a neuroscience study –

Human motor behavior encompasses learning, controlling, and executing actions such as locomotion, posture, tool use, and facial expression. The production and execution of these actions involve the recruitment of multiple complementary systems so that sensory information about our body and environment can be incorporated into our central and peripheral nervous systems. For example, when performing an everyday task, such as reaching for a light switch, we utilize our sensory systems to extract task-relevant information, such as the position of our hand and the distance of the switch. This information is then used to establish our current state relative to the given goal and, via comparison with our previous motor experiences, can be used to formulate a motor plan. Which will then be used to generate a motor command that specifies the required muscular forces to be produced to execute the action successfully.

Our ability to produce these behaviors is continuously evolving throughout our entire lives[15], but the critical phase of sensorimotor development occurs during infancy and early childhood. Here children progressively develop new motor proficiencies such as rolling over by six months and walking by 18 months, allowing them to better to interact with their environment through perception, cognition, and social interaction[16]. Consequently, the child learns the basic three controls: predictive or feed-forward control, reactive control, and bio-mechanical control[17–19].

Contrarily, children diagnosed with a neurological disorder later in life often show differences in motor function, atypical behavioral features, and delays in cognitive development during the sensorimotor development phase[20,21]. For this reason, the Center for Disease Control and Prevention (CDC) has recently devised a new developmental screening process that advises to periodically (9, 18, and 30 months) evaluating children for any developmental delays[22,23]. Generally, motor dysfunctions can be categorized as delays in motor activities, the appearance of atypical motor patterns that affect fine (reaching and grasping) and gross (supine, prone) motor skills, motor stereotypies (repetitive banging), and impairments in bilateral coordination. These all lead to spatial (incorrect body positioning) and temporal (poor movement timing, increased time to initiate movement) disorder[21,24–30].

The evaluation of sensorimotor behavior during childhood often relies on standardized tools, such as the movement assessment battery for children-2 (MABC-2)[31], where an observer (trained practitioner, parent, etc.) scores the motor skills of the child against predefined criteria. Whilst many of these tools have been shown to have high validity alongside high test-retest reliability[31], they do not necessarily allow for the tracking of subtle variations in movement kinematics, which provide invaluable insights into motor control processes beyond the outcome measures of motor performance within these tools. In this area, advances in motion capture technology can be leveraged to bring further objectivity to sensorimotor evaluation for both standardized assessment tools and during natural behavior,[32,33].

We can infer much about child's cognitive states and ongoing decision-making processes just from the kinematics of their movements[34]. A growing number of laboratory studies are now using motion capture and detailed kinematic analyses to investigate questions related to social action and interaction in both typical[35], and atypical populations[36–38]. These experiments, however, are bound to a laboratory.

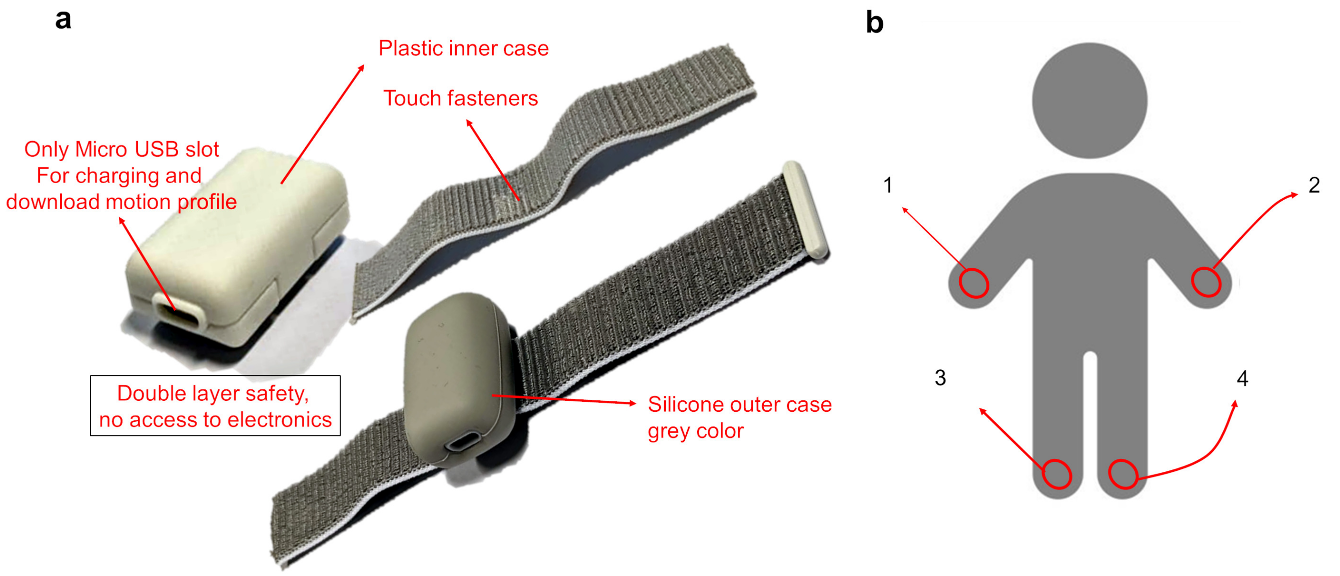

Supplementary Figure 7. a, Kinematics Detector with two-layer protection, first the plastic casing in which electronics are secured, and second the silicone outer layer encapsulates the plastic case to prevent the toddler's access. **b**, The identified positions for wearing KiD.

Supplementary Discussion: Safety measures on wearables design for toddlers

Safety factor recommendations while designing wearable devices for toddlers are a crucial design criterion, (i) Neutral color: Children beyond four months naturally tend to attract colors. Hence, a neutral color must be chosen to discourage the child's attention. (ii) Size: Wearables should be conventionally larger than the children's typical esophagus[39]. Moreover, there should be a safety hole for allowing respiration in case of an accident. (iii) Accessories: Wearables should not have removable accessories (e.g., access media or battery). (iv) Material: Wearables must be made of materials that are devoid of allergens, strong, and resistant to wear and tear.

Kinematics Detector (KiD), by default, has two-layer protection, a silicone outer cover has a particular color (i.e., KiD is grey colored following a neutral color policy) to discourage the child's attention and a `Plastic inner case` to limit the circuitry access to the child[2], as shown in Fig. 7a. This protective outer layer is meant to be larger than a children's typical esophagus[39] to avoid accidental swallowing. Moreover, KiDs do not need external media storage, such as a Secure Digital card for storing motion profiles, to avoid removing the media after every usage. Instead, we have designed KiDs with onboard memory to save motion profiles. KiD provides straightforward wearability (volume of $35\times20\times10\,\mathrm{mm}^3$, and weight 10 g) so that the devices can be worn on the toddlers' limbs (e.g., wrist, arm, leg, torso, and ankle, see the identified at positions 1, 2, 3, and 4 in Fig. 7b) for capturing the associated motion profile. Considering safety factors, short-lived experiments are advisable for toddlers, and hence a battery capacity of 100 mAh can be enough for a simple neurological experiment among toddlers.

**PARAMETERS**	**WITMOTION WT901BLECL**	**3SPACE BLUETOOTH Mini**	**PERCEPTION NEURON 3**	**XSENSE DOT**	**This work KiD**	***Particulars***
DIMENSIONS	51.3 mm X 36 mm X 15 mm	37 mm X 22 mm X 10 mm	43 mm X 33 mm X 20 mm	36. 3mm X 30.5mm X 10.8mm	**35 mm X 20 mm X 10 mm**	The highly compact size and lightweight make our proposal suitable for unobtrusive measurement in small children to old people.
WEIGHT	20 g Without strap	9 g	15.8 g	11.2 g Without strap	**10 g Without strap**	
BLUETOOTH	BLE 5	BLE 4.0	Wireless	BLE 5	**BLE 4.2**	
BATTERY TIME	10 hrs	1.5+ hours	≥3.5 hours	8 hrs	**≥2.5 hours**	Size and weight constraints limit the battery capacity. The battery time of KiD is still enough for the seamless operation during a typical recording session.
BATTERY RATING	260 mAh	-	-	70 mAh	**100 mAh**	
ADVANCED FILTERING	Yes	Yes	-	Yes	**Yes**	Filtering methods to eliminate noise (configurable to obtain raw data or filtered data)
CONNECTION TO PC	USB C	USB 2.0	USB 2.0	USB 2.0	**USB 2.0**	A transfer medium to transfer data to PC, charging the device battery, and reconfigure the device settings (update firmware).
CONFIGURABLE FIRMWARE	-	Yes	-	No	**Yes**	
MAC ID	Yes Bluetooth	Yes Bluetooth	Yes Wireless	Yes Bluetooth	**Yes Bluetooth**	A unique MAC-ID by default to provide device authenticity.
STATUS LED	Yes	Yes	Yes	Yes	**Yes**	To represent the device status for charging, connectivity and operation
BUTTON	Yes	Yes	Yes	Yes	**Yes**	To physically, start and stop the operation cycle.
EXTERNAL CASE	Yes	Yes	Yes	Yes	**Yes**	The protective silicone external case is designed to: i) ensure that our proposal is comfortable and non-distracting during motor performance, ii) cover any light that might distract children, iii) enable immediate technical intervention in case of electronic failure during data collection.
ACCELEROMETER RANGE	±16 g	±2g / ±4g /±6g/ ±8g Selectable	±16 g	±16 g	**±4g / ±8g Configurable**	Smaller range (±4g) helps our proposal to capture the more sensitive information like fine motor skills methodically.
GYRO SCALE	±2000 º/s	±250/±500/ ±1000/ ±2000 º/sec Selectable	±2000 º/s	± 2000°/s	**±2000 º/sec (Maximum) Configurable**	
COMPASS SCALE	±4900 µT	±0.88 Ga to ±8.1 Ga Selectable (±1.3 Ga default)	-	±8 Ga	**±4912µT (Maximum) Configurable**	
INTERNAL STORAGE	-	-	-	64 MB	**32 MB**	The storage space to temporarily store the data inside the device.
USER INTERFACE	Yes	Yes	Yes	Yes	**Yes**	
PLATFORM	Windows	-	-	Windows, Android, IOS	**Windows, Linux, MAC**	
SYNCHRONIZATION	Yes	Yes	Yes	Yes	**Yes**	The UI can send commands concurrently to all the connected devices to have a mutually synchronized data output.
NUMBER OF DEVICES	-	-	5	5	**8**	Number of devices that can run in synchronously.
TRANSCEIVER	-	-	Yes	-	**No**	Uses inbuilt BLE, thus no transceiver is required.
DATA ENCRYPTION	-	-	-	-	**Yes**	Data encryption protects data confidentiality to converting it to an encoded information.
DATA FREQUENCY	0.2 Hz to 50 Hz	475 Hz	-	120 Hz	**200 Hz**	The frequency the data provides the synchronized kinematics data.
LATENCY	-	-	< 20 ms	30 ms	**7.5 ms**	Lesser latency makes the system more responsive, faster and reliable. For instance, The time between START command to the data acquisition start.
RELATIVE PRICE/ COST	-	1.75	2.91	1.094	1*#	* The cost listed is for the prototypes used in this work. However cost can further decrease with larger production volumes<br># Includes the complete electronics fabrication without mechanical parts.

Supplementary Figure 8. Parameter comparison of KiD with other devices available in the market.

Supplementary Methods: Implementation technique

The manuscript's software synchronizes Kinematic Detectors, but to support other devices, the wearable devices must have the following commands (see Supplementary Table 1) in their device functioning. These devices must also promptly provide an acknowledgment (`OK, ERR` along with a timestamp) for post-processing. To evaluate the performance of the local system and devices, the event time from the local system for sending data (`sdelta_t`), receiving data back in the local system (`rdelta_t`), and device time (`ddelta_t`) can be used.

Supplementary References

1. Lee, J., Kwon, J. & Kim, H. Reducing smartwatch users' distraction with convolutional neural network. *Mob. Inf. Syst.* **2018**, 7689549 (2018).
2. Cavallo, A. *et al.* A low-cost stand-alone platform for measuring motor behavior across developmental applications. *iScience* **24**, 102742 (2021).
3. Liu, S., Zhang, J., Zhang, Y. & Zhu, R. A wearable motion capture device able to detect dynamic motion of human limbs. *Nat. Commun.* **11** (2020).
4. Coviello, G. & Avitabile, G. Multiple Synchronized Inertial Measurement Unit Sensor Boards Platform for Activity Monitoring. *IEEE Sensors J.* **20**, 8771–8777 (2020).
5. Bideaux, A., Zimmermann, B., Hey, S. & Stork, W. Synchronization in wireless biomedical-sensor networks with Bluetooth Low Energy. *Curr. Dir. Biomed. Eng.* **1**, 73–76 (2015).
6. Kugler, P., Schlarb, H., Blinn, J., Picard, A. & Eskofier, B. A wireless trigger for synchronization of wearable sensors to external systems during recording of human gait. In *Proceedings of the Annual International Conference of the IEEE Engineering in Medicine and Biology Society, (EMBS)*, 4537–4540 (IEEE, 2012).
7. ST Microelectronics, STM32L476 Datasheet. https://www.st.com/resource/en/datasheet/stm32l476je.pdf (2020).
8. Silicon Labs, Bluetooth Low Energy, BGM123A Datasheet. https://www.silabs.com/documents/public/data-sheets/bgm12x-datasheet.pdf (2020).
9. Bluetooth specification version 4.2. https://www.bluetooth.com/specifications/specs/ (2020).
10. Silicon Labs Bluetooth LE Documentation. https://docs.silabs.com/bluetooth/4.0/general/overview/ (2020).
11. Texas Instruments, Battery controller, BQ24230 Datasheet. https://www.ti.com/product/BQ24230 (2020).
12. Invensense, Inertial Measurement Unit, IMU9250 Datasheet. https://invensense.tdk.com/wp-content/uploads/2015/02/PS-MPU-9250A-01-v1.1.pdf (2020).
13. Flash memory Datasheet. https://eu.mouser.com/datasheet/2/196/CYPR_S_A0011122469_1-3004714.pdf (2020).
14. Balasubramanian, K. K. *et al.* A transcutaneous fetal visual stimulator. *IEEE Access* **10**, 45979–45996 (2022).
15. Adolph, K. E. & Franchak, J. M. The development of motor behavior. *Wiley Interdiscip. Rev. Cogn. Sci.* **8**, e1430 (2017).
16. Malina, R. M. Motor Development during Infancy and Early Childhood: Overview and Suggested Directions for Research. *Int. J. Sport Heal. Sci.* **2**, 50–66 (2004).
17. Wolpert, D. M., Diedrichsen, J. & Flanagan, J. R. Principles of sensorimotor learning. *Nat. Rev. Neurosci.* **12**, 739–751 (2011).
18. Tam, W.-k., Wu, T., Zhao, Q., Keefer, E. & Yang, Z. Human motor decoding from neural signals: a review. *BMC Biomed. Eng.* **1**, 1–22 (2019).
19. Taniguchi, T. *et al.* Symbol Emergence in Cognitive Developmental Systems: A Survey. *IEEE Transactions on Cogn. Dev. Syst.* **11**, 494–516 (2019).
20. Simion, F., Regolin, L. & Bulf, H. A predisposition for biological motion in the newborn baby. In *Proceedings of the National Academy of Sciences of the United States of America*, 809–13 (2008).
21. Bhat, A. N., Landa, R. J. & Galloway, J. C. Current perspectives on motor functioning in infants, children, and adults with autism spectrum disorders. *Phys. Ther.* **91**, 1116–1129 (2011).
22. Estes, A. *et al.* Behavioral, cognitive, and adaptive development in infants with autism spectrum disorder in the first 2 years of life. *J. Neurodev. Disord.* **7** (2015).
23. Developmental Monitoring and Screening for Health Professionals. https://www.cdc.gov/ncbddd/childdevelopment/screening-hcp.html (2021).

24. Esposito, G., Venuti, P., Apicella, F. & Muratori, F. Analysis of unsupported gait in toddlers with autism. *Brain Dev.* **33**, 367–373 (2011).

25. Taffoni, F. *et al.* Sensor-based technology in the study of motor skills in infants at risk for ASD. In *Proceedings of the IEEE RAS and EMBS International Conference on Biomedical Robotics and Biomechatronics*, 1879–1883 (IEEE, 2012).

26. Rad, N. M. *et al.* Stereotypical Motor Movement Detection in Dynamic Feature Space. In *IEEE International Conference on Data Mining Workshops, (ICDMW)*, 487–494 (IEEE, 2016).

27. Bo, J., Lee, C. M., Colbert, A. & Shen, B. Do children with autism spectrum disorders have motor learning difficulties? *Res. Autism Spectr. Disord.* **23**, 50–62 (2016).

28. Foster, N. *Sensorimotor learning and control in autism spectrum disorders: The role of sensorimotor integration.* Ph.D. thesis, Liverpool John Moores University (2018).

29. Yang, X. *et al.* Integrating image and textual information in human–robot interactions for children with autism spectrum disorder. *IEEE Transactions on Multimed.* **21**, 746–759 (2019).

30. Gandotra, A. *et al.* Fundamental movement skills in children with autism spectrum disorder: A systematic review. *Res. Autism Spectr. Disord.* **78**, 101632 (2020).

31. Griffiths, A., Toovey, R., Morgan, P. E. & Spittle, A. J. Psychometric properties of gross motor assessment tools for children: A systematic review. *BMJ Open* **10**, 1–14 (2018).

32. Bisi, M. C., Pacini Panebianco, G., Polman, R. & Stagni, R. Objective assessment of movement competence in children using wearable sensors: An instrumented version of the TGMD-2 locomotor subtest. *Gait & Posture* **56**, 42–48 (2017).

33. Lander, N., Nahavandi, D., Mohamed, S., Essiet, I. & Barnett, L. M. Bringing objectivity to motor skill assessment in children. *J. Sports Sci.* **38**, 1539–1549 (2020).

34. Becchio, C., Koul, A., Ansuini, C., Bertone, C. & Cavallo, A. Seeing mental states: An experimental strategy for measuring the observability of other minds. *Phys. Life Rev.* **24**, 67–80 (2018).

35. Turri, G. *et al.* Decoding social decisions from movement kinematics. *iScience* 105550 (2022).

36. Cavallo, A. *et al.* Identifying the signature of prospective motor control in children with autism. *Sci. Reports* **11**, 1–8 (2021).

37. Foster, N. C. *et al.* Atypical biological kinematics are represented during observational practice. *J. Exp. Psychol. Hum. Percept. Performance.* **44**, 842–847 (2018).

38. Montobbio, N. *et al.* Intersecting kinematic encoding and readout of intention in autism. *Proc. Natl. Acad. Sci.* **119**, e2114648119 (2022).

39. Bott, T. S. *et al.* Esophageal Diameters in Children Correlated to Body Weight. *Eur. J. Pediatr. Surg.* **29**, 528–532 (2019).
