# Supplementary material for: Neural network-based Bluetooth synchronization of multiple wearable devices
Source: Nat Commun. 2023 Jul 25;14:4472. doi: 10.1038/s41467-023-40114-2 (PMC10368670; doi:10.1038/s41467-023-40114-2)
Supplement: Supplementary file 3 — Description of Additional Supplementary Files [file 41467_2023_40114_MOESM3_ESM.pdf]

**Description of Additional Supplementary Files**

File Name: Supplementary Code

Description: Shows how to interface the wearable devices, carry out experiments, and gather data from the local system.
